# Enterobacter cloacae Infection of a Cardiovascular Implantable Electronic Device (CIED): A Rare Cause of Device-Related Infection

**DOI:** 10.7759/cureus.94799

**Published:** 2025-10-17

**Authors:** Sarah Baroud, Nassim Salamin, Rasha Awawdeh, Fuad Alsaraj

**Affiliations:** 1 Internal Medicine, Mediclinic Parkview Hospital, Dubai, ARE; 2 Internal Medicine, Mohammed Bin Rashid University of Medicine and Health Sciences, Dubai, ARE; 3 Internal Medicine/Endocrinology, Mediclinic Parkview Hospital, Dubai, ARE

**Keywords:** cardiac implantable electronic device, device explantation, enterobacter cloacae, infective endocarditis, pacemaker

## Abstract

Cardiac implantable electronic device (CIED) infections are uncommon but serious complications that can be difficult to diagnose, especially in the absence of echocardiographic findings. As CIED utilization increases, so does the risk of infection, underscoring the importance of early recognition and timely management.

We describe a case of *Enterobacter cloacae* (*E. cloacae*) CIED infection, a rarely reported cause of device-related infection, presenting as persistent bacteremia despite susceptibility-guided antibiotic therapy. Repeated echocardiography and advanced imaging failed to localize the source, and management was complicated by inducible AmpC β-lactamase resistance, requiring escalation to carbapenem therapy. The diagnosis was ultimately confirmed by culture of the extracted pacemaker lead tip, with clinical resolution following device removal.

This case highlights the diagnostic and therapeutic challenges posed by atypical *E. cloacae* CIED infections, including imaging limitations and antimicrobial resistance. It emphasizes the value of device-led culture and timely removal in achieving successful outcomes.

## Introduction

Infective endocarditis (IE) is an infection of the endocardial surface, and it most commonly involves native or prosthetic heart valves, the mural endocardium, septal defects, or indwelling cardiac devices [1]. It remains a significant diagnostic and therapeutic challenge. Although it can occur across all age groups, IE carries high morbidity and mortality, particularly when diagnosis is delayed or complications develop. The estimated incidence in the general population is approximately four cases per 100,000 person-years, rising to 15 per 100,000 among individuals over 50 years of age [2]. In the United States, the American Heart Association reports an estimated 10,000 to 15,000 new cases annually [3].

The epidemiology of IE has evolved in recent decades due to an aging population and the increasing prevalence of congenital heart disease, immunosuppression, hemodialysis dependence, and intravenous (IV) drug use [4]. Consequently, prosthetic valve endocarditis and cardiac device-related IE have become more common [5]. The most frequently implicated organisms include *Viridans streptococci, Streptococcus bovis*, the *HACEK* group (*Haemophilus*, *Aggregatibacter*, *Cardiobacterium*, *Eikenella*, and *Kingella*),* Staphylococcus aureus*, and community-acquired enterococci. In contrast, non-HACEK gram-negative bacilli such as *Enterobacter cloacae* (*E. cloacae*) are rare causes of IE, with only a limited number of cases reported in the literature [6].

Against this background, we present the case of a 66-year-old woman with persistent* E. cloacae* bacteremia ultimately traced to an infected pacemaker, despite repeatedly negative echocardiography and advanced imaging. This case illustrates the diagnostic challenges associated with *E. cloacae* cardiac device infections, which remain uncommon and underrepresented in the literature. Persistent bacteremia was the only initial clue until culture of the extracted right ventricular pacemaker lead tip confirmed the diagnosis. The case also highlights the therapeutic challenge posed by inducible AmpC β-lactamase resistance, which required escalation to carbapenem therapy. Clinical resolution occurred only after device removal, highlighting the role of device extraction in the management of cardiac implantable electronic device (CIED) infections.

## Case presentation

A 66-year-old Jordanian woman presented to the emergency department with a three-day history of fever accompanied by chills, night sweats, fatigue, loss of appetite, and generalized body aches. She also reported nausea and a single episode of vomiting and diarrhea but denied abdominal pain or gastrointestinal bleeding (melena, hematochezia, or hematemesis). The remainder of her review of systems was unremarkable, with no respiratory, cardiovascular, neurological, genitourinary, or dermatological symptoms.

Her medical history included hypertension, dyslipidemia, hypothyroidism, anemia of chronic disease, Crohn’s disease in remission, and a prior minor stroke without residual deficits. At age 51, she underwent bioprosthetic aortic valve replacement for severe aortic regurgitation, followed by repeat aortic valve surgery at age 63 for *Brucella* endocarditis. Seven months prior to this presentation, she received a dual-chamber pacemaker implantation for symptomatic bradycardia, which had been functioning normally at her most recent follow-up six months earlier. Long-term medications included azathioprine, mesalamine, amlodipine/valsartan, atorvastatin, levothyroxine, folic acid, and aspirin.

On examination, she was alert, oriented, and not in distress. Her temperature was 38.2°C; other vital signs were stable: heart rate 89 bpm (regular), blood pressure 138/53 mmHg, respiratory rate 16/min, and oxygen saturation 96% on room air. Body mass index was 22 kg/m². Pale conjunctiva and nailbeds were noted. Cardiac auscultation revealed a systolic murmur over the aortic area without signs of heart failure or aortic regurgitation. There were no peripheral stigmata of IE or features of vasculitis. Lung, abdominal, and musculoskeletal examinations were unremarkable aside from osteoarthritic changes.

Admission laboratory tests (Table 1) showed anemia, a normal white blood cell count with neutrophilic predominance, and markedly elevated inflammatory markers: C-reactive protein at 178 mg/L, erythrocyte sedimentation rate at 68 mm/hr, and procalcitonin at 45 ng/mL. Ferritin was at the upper limit of normal, and mild troponin I elevation and hypokalemia (3.0 mmol/L) were also noted.

**Table 1:** Laboratory results on admission eGFR: estimated glomerular filtration rate, AST: aspartate aminotransferase, ALT: alanine aminotransferase, ALP: alkaline phosphatase, LDH: lactate dehydrogenase, APTT: activated partial thromboplastin time, ESR: erythrocyte sedimentation rate, RBS: random blood sugar

Lab markers	Result	Reference
White blood count	9.2 10^3 ^uL	4-11.0 10^3 ^uL
Neutrophils	80.45%	43.5-73.5%
Lymphocytes	14.66%	15.20-43.30%
Eosinophils	0.02%	0.8-8.10%
Monocytes	4.36%	5.50-13.70%
Basophils	0.31%	0.2-1.5%
Red blood count	3.69 uL	4.50-5.90 10^6 ^uL
Hemoglobin	10.0 g/dl	13.0-17.5 g/dl
Hematocrit	30.2%	37.0-47.0%
Mean corpuscular volume	81.8 fl	80.0-100.0 fl
Ferritin	116 ng/mL	30.00-120.00 ng/mL
Platelet count	216 10^3 ^uL	150-450 10^3 ^uL
Sodium	140 mmol/L	136-145 mmol/L
Potassium	3.0 mmol/L	3.5-5.10 mmol/L
Bicarbonate (HCO3)	30 mmol/L	22-28 mmol/L
Chloride	97 mmol/L	98-107 mmol/L
Corrected calcium	2.46 mmol/L	2.20-2.55 mmol/L
Inorganic phosphate	1.17 mmol/L	0.74-1.52 mmol/L
Magnesium	0.73 mmol/L	0.66-1.07 mmol/L
C-reactive protein	178 mg/L	0-5 mg/L
Procalcitonin	45 ng/mL	Normal: <0.05 ng/mL
ESR	68 mm/hr	3-55 mm/hr
Creatinine	94.4 umol/L	62.0-106.0 umol/L
Urea	4.1 mmol/L	3.5-7.2 mmol/L
eGFR	54 ml/min /1.73m2	>60 ml/min
Bilirubin total	18.40 umol/L	<21 umol/L
AST	32 U/L	<50 U/L
ALT	28 U/L	<50 U/L
Total protein	77 g/L	58-76 g/L
Albumin	36.1 g/L	32-46 g/L
ALP	198 U/L	40-129 U/L
Troponin	0.02 Ng/ml	0.0000-0.0156 Ng/ml
RBS	6.75 mmol/L	3.89-7.7 mmol/L
LDH	232 U/L	125-243 U/L
APTT control	32.0 seconds	28.0-40.0 seconds

Urinalysis revealed mild proteinuria (+1). Complement levels (C3, C4) were normal, and troponin normalized on repeat testing. The initial infectious workup, including respiratory viral panel, COVID-19 PCR, urine culture, and *Brucella* IgM serology, was negative.

A 12-lead electrocardiogram (ECG) demonstrated atrial sensing with ventricular pacing. A chest X-ray showed a dual-chamber pacemaker in situ, aortic calcification, and the aortic valve sewing ring, with clear lung fields. Computed tomography (CT) of the chest revealed no pulmonary consolidation, pleural effusion, mediastinal lymphadenopathy, or masses. Transthoracic echocardiography (TTE) showed a thickened aortic valve with a small paravalvular leak but otherwise normal bioprosthetic valve function, preserved left ventricular function, no vegetations, and a pacemaker lead in the right ventricle.

Three sets of blood cultures were obtained at hourly intervals according to hospital protocol, with each set consisting of an aerobic and anaerobic bottle. Empirical IV ceftriaxone (2 g daily) was initiated. Azathioprine was withheld, potassium supplementation was started, and thromboembolic deterrent stockings were applied for deep vein thrombosis prophylaxis. Additional monitoring included daily ECGs for IE-related changes (e.g., new heart block or ischemia).

The patient partially improved after 72 hours, with reduced fever and fewer chills. Daily examinations remained negative for signs of IE or heart failure. All three initial sets of blood cultures grew pan-sensitive *E. cloacae* in both aerobic and anaerobic bottles. Transesophageal echocardiography (TEE) revealed no vegetations, masses, or thrombi on the valves, left atrial appendage, or right ventricular pacemaker lead. Given these findings, a gastrointestinal source was considered; however, stool studies, contrast-enhanced CT of the abdomen and pelvis, and colonoscopy were unrevealing aside from mildly active Crohn’s colitis.

In view of the culture results, IV gentamicin was added to ceftriaxone (160 mg loading dose, then 80 mg (1 mg/kg) every eight hours) for potential synergistic bactericidal activity. Although robust data supporting gentamicin use in non-HACEK gram-negative endocarditis are limited, combination β-lactam-aminoglycoside therapy is consistent with IE treatment guidelines. It has been described in similar *E. cloacae* IE cases with favorable outcomes. The regimen was chosen in consultation with the infectious disease (ID) team, with close monitoring for toxicity and emerging resistance. The ID team recommended two weeks of gentamicin with ceftriaxone, followed by ceftriaxone plus ciprofloxacin (400 mg IV every 12 hours) for an additional four weeks. Antibiotics were administered via peripheral cannulas, which were rotated every 72 hours.

Despite 10 days of ceftriaxone and six days of gentamicin, bacteremia persisted, and the patient’s clinical condition remained unchanged. Repeat TTE showed no new findings. Given concern for inducible AmpC β-lactamase resistance, ceftriaxone was replaced with IV ertapenem. The next set of blood cultures, the first to return negative, was reported after five days of ertapenem therapy (with four days of overlapping gentamicin), confirming clearance of bacteremia after 14 days. The patient demonstrated clinical and biochemical improvement (Table 2), and a peripherally inserted central catheter (PICC) was placed to facilitate outpatient therapy.

**Table 2:** Laboratory results on day 14 of antibiotic therapy (day of hospital discharge)

Lab markers	On admission	Day 3 on antibiotics	Day 10 on antibiotics	Day 14 on antibiotics (discharge day)	References
White blood count	9.2 10^3^ L	4.6 10^3^ μL	10.1 10^3^ μL	5.0 10^3^ μL	4.-11.0 10^3 ^μL
Neutrophils	80.45%	47.77%	64.46%	56.07%	43.5-73.5%
Red blood count	3.69 μL	3.28 μL	3.30 μL	3.49 μL	4.50-5.90 10^6 ^μL
Hemoglobin	10.0 g/dl	8.9 g/dl	8.8 g/dl	9.5 g/dl	13.0-17.5 g/dl
C-reactive protein	178 mg/L	99 mg/L	95.9 mg/L	22.9 mg/L	0-5 mg/L
Procalcitonin	45 ng/mL	29 ng/mL	7 ng/mL	0.9 ng/mL	Normal: <0.05 ng/mL

The patient was discharged to complete a six-week course of IV ertapenem, with the first four weeks overlapping gentamicin, followed by two weeks of oral ciprofloxacin. During outpatient follow-up, she remained afebrile but reported persistent weakness, and laboratory values steadily improved. All subsequent blood cultures remained negative throughout therapy, and serial transthoracic echocardiograms performed every two weeks demonstrated no interval changes.

Twenty-five days into therapy, she developed acute kidney injury, likely secondary to gentamicin-induced acute tubular necrosis, with a possible contribution from valsartan. Both agents were discontinued, and renal function subsequently recovered. Upon completion of six weeks of IV ertapenem, the patient was afebrile and asymptomatic; the PICC line was removed, repeat echocardiography showed no new findings, and laboratory markers were near normal (Table 3).

**Table 3:** Laboratory markers post-AKI and at completion of six weeks of antibiotic therapy eGFR: estimated glomerular filtration rate, AKI: acute kidney injury

Lab markers	Result	Reference
White blood count	6.6 10^3^ μL	4-11.0 10^3^ μL
Neutrophils	60.78%	43.5-73.5%
Red blood count	3.60 μL	4.50-5.90 106 μL
Hemoglobin	9.5 g/dl	13.0-17.5 g/dl
Platelet count	273 10^3^ μL	150-450 10^3^ μL
Sodium	139 mmol/L	136-145 mmol/L
Potassium	3.7 mmol/L	3.5-5.10 mmol/L
C-reactive protein	6.3 mg/L	0-5 mg/L
Procalcitonin	0.05	Normal: <0.05 ng/mL
Creatinine	74.5 μmol/L	62.0-106.0 μmol/L
Urea	4.5 mmol/L	3.5-7.2 mmol/L
eGFR	72 ml/min/1.73 m^2^	>60 ml/min

The plan was to repeat blood tests every two weeks for the first month and then monthly thereafter, with follow-up echocardiograms scheduled at one and three months post-treatment. However, 72 hours after completing antibiotics, the patient was readmitted with fever and elevated inflammatory markers (Table 4).

**Table 4:** Laboratory results at readmission 72 hours after completing antibiotic therapy eGFR: estimated glomerular filtration rate

Lab markers (72 hours off antibiotics)	Result	Reference
White blood count	8.8 10^3^ μL	4.-11.0 10^3^ μL
Neutrophils	81.70%	43.5-73.5%
Red blood count	3.23 μL	4.50-5.90 106 μL
Hemoglobin	8.8 g/dl	13.0-17.5 g/dl
Platelet count	246 10^3^ μL	150-450 10^3^ μL
C-reactive protein	127 mg/L	0-5 mg/L
Procalcitonin	4.95 ng/mL	Normal: <0.05 ng/mL
Creatinine	68 μmol/L	62.0-106.0 μmol/L
eGFR	77 ml/min/1.73 m^2^	>60 ml/min

Repeat blood cultures once more grew *E. cloacae*, which is now resistant to amoxicillin/clavulanic acid, cefepime, cefotaxime, ceftazidime, and piperacillin/tazobactam, but still sensitive to ertapenem and ciprofloxacin. A new PICC was placed, and IV ertapenem (1 g daily) was resumed for a planned six-week course.

A CT-PET scan and leukoscan were performed but did not reveal a source of infection, and given the recent normal transesophageal echocardiogram, repeat imaging was not immediately indicated. Brucella serology and cultures on specific media were also negative. A multidisciplinary discussion was reconvened with the ID and cardiology teams, this time including cardiothoracic surgery, to assess the need for surgical intervention. During this period, the patient’s bacteremia cleared, and she improved clinically. Given the high surgical risk and absence of valvular vegetations, she was discharged to complete her antibiotic course.

After completing six weeks of therapy, the ID team recommended repeating blood cultures after 72 hours. However, within 48 hours, the patient redeveloped a fever, and the cultures again grew *E. cloacae*. Ertapenem was resumed, and with no alternative sources identified, the pacemaker was considered the likely nidus of infection. She was referred to electrophysiology, and the device was subsequently explanted. The culture of the extracted right ventricular pacemaker lead tip grew *E. cloacae*, confirming the device as the source of infection. Two weeks after device replacement, the patient remained afebrile off antibiotics, with no evidence of recurrent bacteremia.

## Discussion

IE is a life-threatening infection of the endocardial surface, most often affecting native or prosthetic valves. Despite advances in diagnostics and therapy, it continues to carry high morbidity and mortality, particularly when complicated by heart failure, embolic events, or persistent bacteremia. Its epidemiology is evolving, with antimicrobial resistance, increasing cardiac surgeries, prosthetic valve implantation, and IV drug use driving incidence. Expanding at-risk groups include adults with congenital heart disease, patients with IV catheters, those on dialysis, immunocompromised individuals, and patients with frequent healthcare exposure [1-4].

*Staphylococcus aureus* remains the predominant pathogen in both community and healthcare settings. IE caused by non-HACEK Gram-negative aerobic bacilli, including *Enterobacteriaceae* and *Pseudomonas* species, is rare. In a large multinational database of 2,761 patients from 61 hospitals across 28 countries, only 49 cases (1.8%) were attributable to non-HACEK Gram-negative bacilli, a finding corroborated by the ICE cohort. Due to this rarity, prospective trial data are lacking to define the optimal antimicrobial regimen. Combination therapy with a β-lactam (penicillins, cephalosporins, or carbapenems) plus an aminoglycoside or fluoroquinolone for approximately six weeks is generally considered reasonable. Early consultation with an ID specialist is recommended, as these organisms may harbor various resistance mechanisms, including inducible β-lactamases. In one study, a case of IE due to *E. cloacae* resistant to third- and fourth-generation cephalosporins was successfully treated with meropenem and amikacin for 12 weeks [5-7].

The widespread use of CIEDs, including pacemakers and implantable cardioverter-defibrillators, has led to a rise in device-related infections, typically classified as pocket infections or lead-associated endocarditis. These infections may result from direct contamination at implantation, hematogenous seeding, or skin erosion. Gram-positive cocci, particularly *Staphylococcus aureus* and coagulase-negative staphylococci, remain the predominant pathogens, while non-HACEK gram-negative bacilli such as *E. cloacae* are rarely reported. Diagnosis is usually straightforward when local pocket inflammation is present, which remains the most common presentation. However, in patients without overt pocket findings, CIED-related IE is often difficult to detect due to nonspecific symptoms and intermittent bacteremia and is associated with high relapse rates when device removal is delayed. Adjunctive imaging modalities, such as TEE, 18F-FDG CT-PET, and nuclear scans (e.g., gallium or leukoscans), may aid in detecting device-related infections but do not always identify the nidus of infection. In such diagnostically challenging cases, standardized definitions, such as the 2023 American Heart Association (AHA) Clinical Definitions of CIED Infection and the Novel 2019 International CIED Infection Criteria, provide valuable guidance in classifying infection likelihood. Preventive strategies, such as preoperative IV antibiotics, careful hemostasis to avoid hematoma formation, and, in high-risk patients, absorbable antibiotic envelopes, are recommended. Management typically requires prolonged IV antibiotics and complete device removal, as eradication without explantation is rarely successful, and delayed or incomplete removal increases morbidity and mortality [5-14].

Our case demonstrates the challenges in diagnosing and managing a CIED infection secondary to *E. cloacae* in an elderly, immunocompromised patient with additional risk factors, including prosthetic valve replacement and pacemaker implantation. Initial treatment with ceftriaxone, later supplemented with gentamicin for a potential synergistic effect, ultimately required escalation to carbapenem therapy due to the emergence of resistance. Despite extensive imaging, including repeated transthoracic echocardiograms, a TEE, CT-PET, and a leukoscan, no clear infectious focus was identified. According to the 2023 AHA Clinical Definitions of CIED [13], the patient initially met the definition of possible CIED infection, based on persistent bacteremia with a nonstaphylococcal organism, absence of an alternative source, and negative TEE findings. The diagnosis was definitively confirmed post-extraction, when culture from the right ventricular pacemaker lead tip yielded *E. cloacae* identical to the blood isolates, fulfilling the criteria for definite CIED infection, and achieving definitive resolution following device removal.

To our knowledge, few cases of *E. cloacae* CIED-related IE have been reported, making this case a rare and instructive example that raises several important considerations in patients with CIED-related infections. Persistent bacteremia should raise suspicion for device-related infection, particularly when multimodal imaging is unrevealing. *E. cloacae*, though uncommon, may harbor inducible AmpC β-lactamase resistance, highlighting the importance of susceptibility-guided therapy. Microbiologic evaluation of extracted hardware can be valuable in establishing a definitive diagnosis when imaging studies are inconclusive. Furthermore, device removal was associated with resolution in this patient and highlights its potential role in managing complex CIED infections. Finally, management of such complex cases typically warrants a multidisciplinary approach, incorporating expertise from relevant specialties, such as ID, cardiology, cardiac surgery, and electrophysiology [5-9,11,13].

## Conclusions

CIED infections are an increasingly recognized clinical challenge as device use expands among aging populations and patients with complex comorbidities. These infections may present without obvious localizing signs or clear echocardiographic findings, with persistent bacteremia sometimes serving as the only clue. Although rare, *E. cloacae* can be a clinically significant cause of device-related infections. Management typically relies on identifying the infectious focus, administering antimicrobial therapy guided by susceptibility, and planning timely device removal. Given the diagnostic challenges and risk of relapse, maintaining a high index of suspicion and using a coordinated multidisciplinary approach can help achieve optimal patient outcomes.
